# Isolation of *Helicobacter pylori* from dental plaque: A microbiological study

**DOI:** 10.4103/0972-124X.44098

**Published:** 2008

**Authors:** Uma Sudhakar, C. N. Anusuya, T. Ramakrishnan, R. Vijayalakshmi

**Affiliations:** *Department of Periodontics, Meenakshi Ammal Dental College, Maher University, Chennai, Tamilanadu, India*

**Keywords:** Carcinogen, culture method, dental plaque, duodenal ulcer and gastric ulcer, *Helicobacter pylori*, rapid urease test, reservoir

## Abstract

**Aim:**

The aim of our study was to isolate *H. pylori* from dental plaque in gastric and duodenal ulcer patients and compare it with dental plaque of healthy subjects.

**Materials and Methods:**

Fifty patients in the age range of 25-50 years who were endoscopically proven cases of duodenal and gastric ulcer were chosen. *H. pylorus* was isolated from the dental plaque of these patients using culture method and rapid urease test (RUT). It was compared with the dental plaque from control group (25 students). The specificity and sensitivity of RUT was compared with culture method. The oral hygiene index (OHI) score and plaque index were assessed.

**Results:**

Ten percent positivity was observed in the study group by culture. Though RUT showed 70% positive isolation it is neither a specific test nor a conclusive test as compared to culture. The result correlates with oral hygiene in study population.

**Conclusion:**

Further, more studies are needed to compare RUT and culture, with serology and polymerase chain reactions. The ability to detect *H. pylori* from dental plaque using these methods offer the potential for the noninvasive test for infection and would aid in support of oral transmission of *H. pylori*.

## INTRODUCTION

The adult human body consists of 10^13^ somatic cells and 10^14^normal or commensal microbes.[1] These commensal bacteria reside on the surfaces of teeth or prostheses within complex ecosystems termed biofilm. Dental plaque is the host-associated biofilm. Supragingival and subgingival plaque provides an optimal aerophilic and microaerophilic environment for the survival of these microorganisms. Approximately six billion microbes representing 300-500 reside in these environments in the oral cavity.

An association between oral infections and systemic diseases has been suspected for centuries. The effect of oral health on the rest of the body was proposed by Assyrians in 7^th^ century BC. In the 18^th^ century, Pennsylvanian physician[1] named Benjamin Rush was quoted as remarking that arthritis could be treated in some people after they had infected teeth extracted.

Over the past decade, a growing body of scientific evidence suggests an exquisite association between oral infection (e.g., viruses, bacteria, yeast) and systemic diseases (e.g., atherosclerosis, cardiovascular disease, cerebrovascular disease, premature low birth weight, and pulmonary diseases and disorders), and also between systemic diseases (e.g., arthritic, diabetic, HIV, and osteoporotic) and oral, dental, and craniofacial disorders.

*H. pylorus* is one such organism which may reside in dental plaque. Area around the dental plaque has a low oxidation potential promoting growth of facultative anaerobes. Newman[2] suggested that bacteria fermenting carbohydrates in food produce a low pH in the dental plaque and this microaerophilic acidic environment with an average oral temperature of 35-37°C can be ideal for growth of *H. pylori*.

*H. pylorus* is a fastidious, microaerophilic, spiral, Gram-negative organism. These bacteria are strongly associated with acute and chronic gastritis as well as with peptic and duodenal ulcers. It has been quite a shock to the medical system to discover that several of the gastroduodenal diseases that scientists thought that they understood fairly well are actually caused by *H. pylori*.

These organisms were first cultured in 1982.[3] The questions that arises in our mind now is, why was this not discovered earlier. Most of our minds were fixed on two simple concepts. The stomach secretes acid to keep it sterile. Too much acid secretion causes ulcers. Gastric acid does kill bacteria, but *H. pylori* have evolved special features which allow them to thrive in this acidic niche. The primary sanctuary of *H. pylori* is the stomach because of its specific binding to gastric cells of the surface.

About 50% of the subjects living in developed and developing countries, by the age of 50 years are affected by *H. pylori*. These infections are particularly difficult to eradicate and it has been postulated that a sanctuary or sanctuaries which allow them to evade antimicrobial therapy much exists. Desai and Majmudar[4] suggested that recrudescence of infection following cessation of therapy may occur, owing to the recolonization of the stomach from the *H. pylori* present in dental plaque which are unaffected by the antimicrobial treatment. Knowledge of this pathogenic organism will permit not only a target for therapeutic procedures but also a monitoring tool for efficacy of therapy and also to learn about the various routes of transmission.

### Aim

The aim of our study was to isolate *H. pylori* from dental plaque in gastric and duodenal ulcer patients and compare it with dental plaque of healthy subjects.

## MATERIALS AND METHODS

The study group consisted of 50 patients, age ranging from 25-50 years, who were endoscopically proven cases of duodenal and gastric ulcer attending the gasteroenterology department of Government Medical College, Chennai. Twenty five students and hospital employees of Madras Medical and Dental College, age ranging from 25-50 years, were taken for the control group. The study group was subdivided into group G, patients with gastric ulcer; group D, patients with duodenal ulcer; and group C, control group.

Subjects with a history of alcoholic abuse, on systemic antibiotics, and chronic use of nonsteroidal anti-inflammatory drugs were excluded from this study. Subjects were briefed about the study and informed consent was obtained.

The study group and control group underwent a gingival evaluation which included measurement of oral hygiene status by simplified oral hygiene index [Greene and Vermilion] and plaque index.[5]

### Sampling of the dental plaque

Dental plaque was removed from the tooth surfaces with a sterile curette. Plaque was collected by an upward scrape against the tooth surface. The sample was dispersed separately in 1 ml of urea broth with phenyl red indicator[6] to detect the urease activity and in 1 ml of normal saline for culture in selective media.

### Isolation procedure

The sample collected in urea broth was monitored for a change in color from orange to pink which is due to the production of urease that converts the urea to ammonia. The broth is monitored for 24 hours. The change occurring within 15-30 minutes denotes a positive reaction.

The sample in saline was transformed to the microbiology lab within 20 minutes at 2-8°C. It was inoculated into the selective media* (CAMPYLOBACTER agar base - Skirrow's medium, High media) and incubated for five days at 37°C in a candle jar which provides the microaerophilic condition.

After five days of incubation the colonies showing the following morphology - circular, convex, translucent, and glistening with slight hemolysis in blood agar - were presumptively identified as *H. pylori*. A Gram stain was then performed on these colonies and was checked for cellular morphology. The colonies demonstrated the characteristic ‘S’ shaped or curved Gram-negative rod appearance. No spores were formed in blood agar culture and spiral forms were less obvious with cells appearing more frequently as singly ensured rods. As the culture aged, the colonies changed from rod to coccoid form.

The following tests were performed to confirm *H. pylori:* rapid urease test (RUT),[6] oxidase test, catalase test, andmotility test.

### Statistical analysis

Chi-square test with Yates correction was employed to test the proportion between the study groups. Student independent *t*-test was employed to compare the mean values between the different study groups. One-way ANOVA was employed when more than two groups were to be compared. To identify the significant group, Turkey HSO procedure was employed after one-way ANOVA.

In the present study, a probability of *P* < 0.05 was considered as the level of significance.

Ninety five percent confidence interval was constructed to obtain the true range of population proportion of organism among ulcer patients. Specificity and sensitivity of the tests were also analyzed.

## RESULTS

*H. pylori* in dental plaque were assessed using two methods. The sensitivity and specificity of RUT was compared with culture which is the gold standard. The significance of the oral hygiene status in these individuals was assessed. Five patients were positive for *H. pylori* by culture. Thirty seven patients were positive by RUT. The 95% confidence interval for the proportion of the patients positive for *H. pylori* was assessed.

The 95% confidence interval is 1.7-18.3%. The *H. pylori* positivity in duodenal and gastric ulcer patients was assessed (*P* = 0.17). It was not significant. Test of significance was assessed. A significant proportion of *H. pylori* is present among the ulcer patients (*P* = 0.02).

[Table 1] shows the assessment of diagnostic tests. RUT method versus culture method for all cases. RUT gives 100% sensitivity whereas specificity is 54.3% which is very low. False-positive rate is 45.7% and false negative is 0% which is encouraging.

**Table 1:** Assessment of diagnostic tests*

RUT	Culture		Total
		
	Positive	Negative	
Positive	5	32	37
Negative	0	38	38
Total	5	70	75

* Population includes study and control groups sensitivity, *n* = 5/5, 100%; specificity, *n* = 38/70, 54.3%; false-positive rate, *n* = 32/70, 45.7%; false-negative rate, *n* = 0/5, 0%

Sensitivity, *n* = 5/5, 100%; specificity, *n* = 15/45, 33.3%; false-positive rate, *n* = 30/45, 66.7%; and false-negative rate, *n* = 0/5, 0%

The sensitivity and specificity of RUT method versus culture method for the patients is presented in [Table 2]. It was found that RUT gives 100% sensitivity but its specificity is 33.3%. The false-positive rate is 66.7% which is very high.

**Table 2:** Assessment of diagnostic tests in case group

RUT	Culture		Total
		
	Positive	Negative	
Positive	5	30	35
Negative	0	15	15
Total	5	45	50

Sensitivity, *n* = 1/1, 100%; specificity, *n* = 10/26, 38.5%; false-positive rate, *n* = 16/26, 61.5%; and false-negative rate, *n* = 0/1, 0%

The proportion of positivity of organisms in groups G and D is shown in [Table 3]. It was found that 17.4% of patients belonging to group 2 had positivity when compared to 3.7% of patients belonging to group C. It was not significant.

**Table 3:** Assessment of diagnostic tests in group G

RUT	Culture		Total
		
	Positive	Negative	
Positive	1	16	17
Negative	0	10	10
Total	1	26	27

Sensitivity, *n* = 4/4, 100%; specificity, *n* = 5/19, 26.3%; false-positive rate, *n* = 14/19, 78.7%; and false-negative rate, *n* = 0/4, 0%.

The comparison of age and OHI-S score in study and control group is presented in [Table 4]. The mean OHI-S score in controls (1.56± 0.68) is significantly lower than group G (3.05 ± 0.58) and group D (3.51 ± 0.58).

**Table 4:** Assessment of diagnostic tests in group D

RUT	Culture		Total
		
	Positive	Negative	
Positive	4	14	18
Negative	0	5	5
Total	4	19	23

Comparison of plaque index score in study group and control group is presented in [Table 5]. Multiple comparison test by Turkey HSO procedure showed a significantly lower plaque index score (*P* < 0.05) in control than the study group. But there was no significant difference between group G and D.

**Table 5:** Proportion of positivity in groups G and D

Culture	Group G	Group D	*P*-value*
Positive	1(3.7%)	4 (17.4%)	0.17 (NS)
Negative	26	19	0.17 (NS)
Total	27	23	-

NS: Non significant,

* Fischer's extract was employed to estimate *P* value.

## DISCUSSION

In his unquenchable thirst for conquering the disease, man has focused his research at the cellular and molecular levels to understand the disease process better. Even since the discovery of *H. pylori* by Marshall and Warren in 1982,[3] its role in gastric pathophysiology represents a fundamental change in the understanding of peptic ulcer diseases.

Parronet in 1998[7] reported that *H. pylorus* was the most common infection in human. The rate of acquisition of *H. pylori* infection was higher in developing than in developed countries. Even within the developed countries, the prevalence varies between ethnic and racial groups and could be due to differences in cultural background, social, and environmental factors. However, the disease only occurs in about 15% of infected persons. The virulence of the strain was the major determinant of who develop the disease. Important virulence factors responsible for the infected strain were thought to include the spiral shape of the bacterium, flagella that allows it to move rapidly, the presence of enzyme urease which buffer gastric acid, presence of adhesive which are site specific gastric epithelium, production of vacuolating cytotoxin, cytotoxin associate gene production A, and finally the ability to stimulate neutrophils to degranulate. The genetic susceptibility of the host also influences the cause of the disease.

Two main mechanisms were suggested by which *H. pylori* may produce gastric inflammation.[8] The organism may interact with surface epithelial cells, producing either direct cell damage or liberation of epithelial proinflammatory mediators; and *H. pylori* products may gain access to the underlying mucosa, thereby directly stimulating host nonspecific and specific immune responses involving the liberation of variety of cytokines (TNFα, IL, IL-6, IL-7, IL-10, and IL-12). Direct mucosal damage may be due to adherence of the organisms to the gastric epithelium, vacuolating cytotoxin which include vacuole formation in epithelial cells, variety of enzymes like urease, which by producing ammonia not only protect the organisms from gastric acid but also have toxic effects on the mucosa, and bacterial phospholipase that degrade the phospholipids components of gastric mucosal barrier.

*H. pylori* is the first bacterial infection recognized as the human carcinogen (National institute of Health Consensus Development).[9] It is also associated with gastric carcinoma and MALT syndrome, asthma, cerebrovascular, and cardiovascular diseases.

*H. pylori* infections are particularly difficult to eradicate. To control the infection there is need to know about the routes of entry and various reservoirs. Various modes of transmission like oro-oral, feco-oral, and spread by water and through food have been implicated.

Dental plaque has been implicated as a possible source and route of transmission of *H. pylori*.[10] Krajen *et al*,[11] first reported on the presence of *H. pylori* in dental plaque. Subsequently, various studies reported a wide range of isolation. These variations may reflect on the methods used, technical difficulties, microbiotic complexes, geographic distribution, and host response. Dental plaque can be defined as the soft deposits that form the biofilm adhering to the tooth surface or other hand surfaces in the oral cavity including removable and fixed restorations. The significance of the biofilm environment has been increasingly recognized in recent years because the environment itself may alter the properties of the organisms. The biofilm community is initially formed through bacterial interactions with the tooth and then through physical and physiologic interactions among different species within the microbial mass. Newer microscopic technique reveals that plaque is actually heterogenous in structure, with clear evidence of open fluid-filled channels running through the plaque mass.[11]

**Figure 1:**
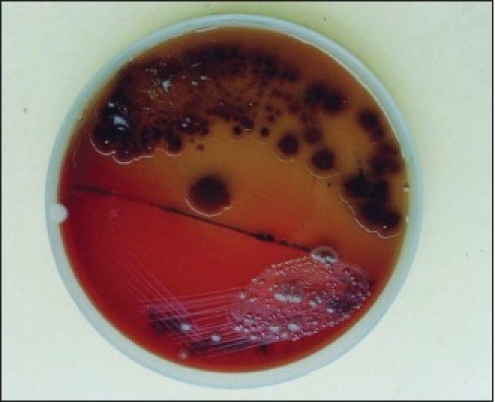
Photograph of culture plate showing *Helicobacter pylori* and Pseudomonas

**Figure 2:**
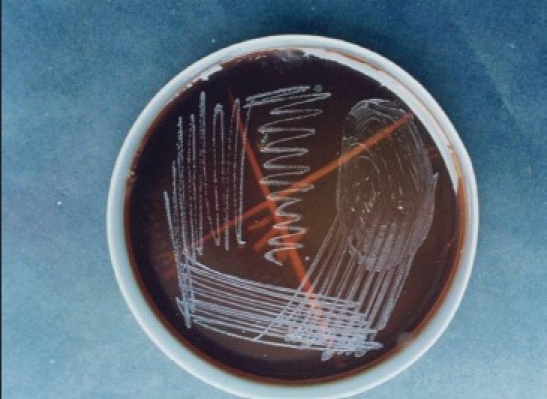
Photograph of subculture of *Helicobacter pylori*

**Figure 3:**
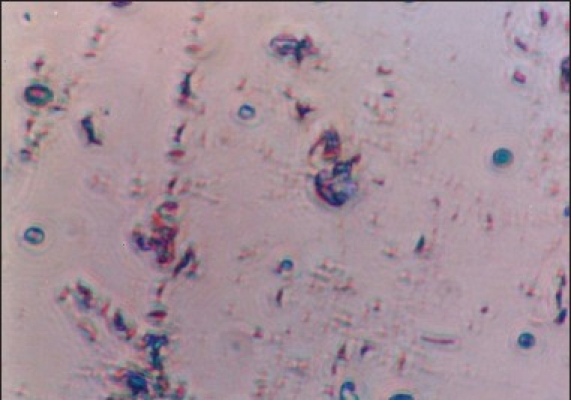
Photomicrograph showing *Helicobacter pylori* colonies

**Figure 4:**
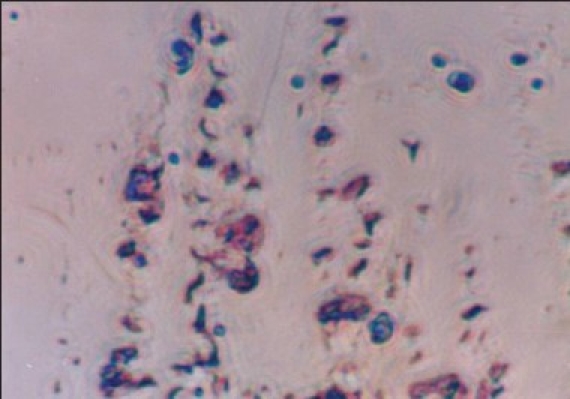
Photomicrograph showing typical ‘S’ shaped colonies of *Helicobacter pylori*

**Figure 5:**
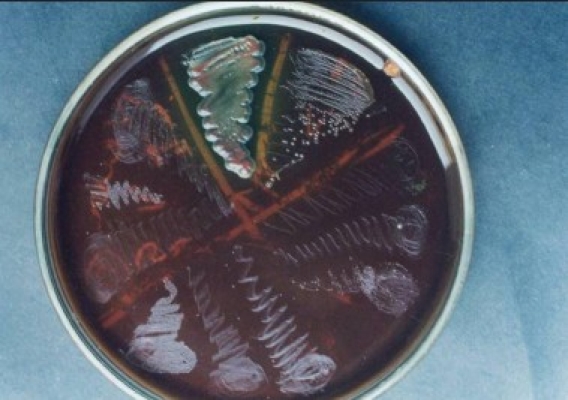
Positive isolates of *Helicobacter pylori*

These channels may provide for circulation within plaque to facilitate movement of soluble molecules such as nutrients or waste products. This biofilm matrix acts as barrier. Substances produced by bacteria within the biofilm are retained within the biofilm. The resistance of bacteria to antimicrobial agents is significantly increased in a biofilm environment. This may be related to the limited diffusion of substances into the biofilm matrix, the slow rate of cell growth in the biofilm environment, and possibility to alter properties of bacteria in response to growth on a surface.

In our study, two methods - RUT and culture - were used for isolation of helicobacter pylori from dental plaque. The specificity and sensitivity of RUT was compared with that of culture. By culture, *H. pylori* were detected in 10% of the patients. The results were similar to the one obtained by Khandeker[12] and Namavan.[13] There was variation in recovery rate of oral *H. pylori* from 0-100% in various studies reported since then. Banatwala[14] reported on a 30% isolation. Humar Quershi[15] reported on 50% isolation. Bernander,[16] and Hardo[17] reported on no isolation from dental plaque. These variations could be due to the insufficient number of organisms, the presence of unculturable, but a viable coccoid forms in polymicrobial oral specimens.[18] The other reasons may be the sensitivity of the culture media or the usage of systemic antibiotics before sampling which may have resulted on the loss of microorganisms.

**Figure 6:**
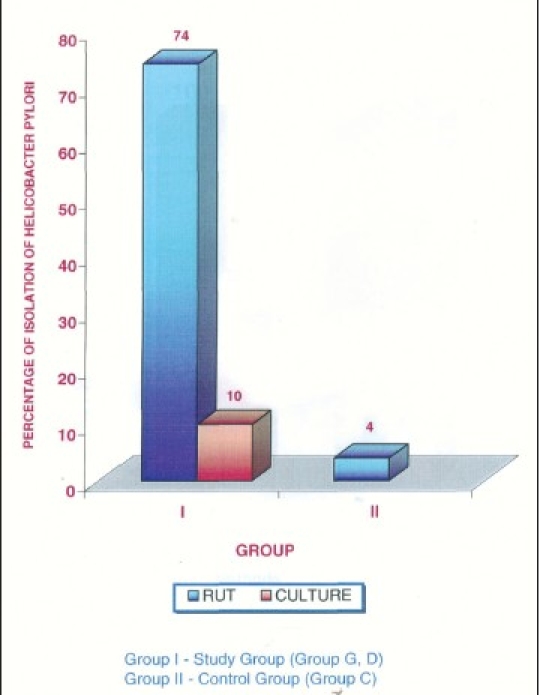
Percentage of isolation of *Helicobacter pylori*

**Figure 7:**
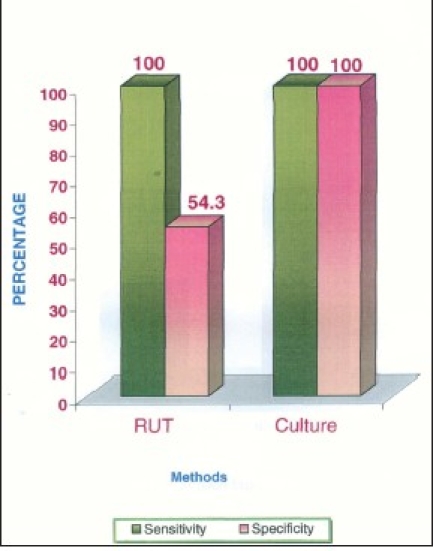
Sensitivity and specificity of isolation methods

In our study, RUT was positive in 70% of the study group and 10% of the control group. This was similar to the one reported by Anne Marie, 1995.[10] In the present study, the sensitivity of RUT was 100% and the specificity was only 54.8% and the false-positive rate was 45.7%. But it was observed that the positive RUT from plaque within 10-15 minutes showed higher culture positivity. This suggests that the specificity of RUT can be increased when the time duration is restricted to 10-15 minutes. This would be due to the presence of other urease-producing organisms like streptococcus spp., *Bacteroides ureolyticus*, and Actinomyces spp. which are found as a part of normal oral flora. Ten percent positivity in controls may be due to these organisms. For this reason, RUT cannot be recommended for identification of *H. pylori*. In the present study, OHI and plaque index scores were assessed between study and control groups, OHI-score in controls was 1.56 which was significantly lower than the study group's value of 3.05 (*P* < 0.001). The mean plaque index score of control group is significantly lower than in study groups. This result was different from some studies which could be due to variations in socioeconomic status, awareness of oral hygiene, and dietary habits in India.[10]

Antimicrobial therapy frequently failed to cure *H. pylori* infections. This may be due to sanctuary sites where the organisms reside. One such site may be the oral cavity. The mechanism by which *H. pylori* reaches the oral cavity is unknown. Anne Marie in 1995[10] reported on the occasional reflex of *H. pylori* from the gastric reservoir leading to colonization of the oral cavity, which may be true. In our study, 10% of patients showed *H. pylori* from dental plaque by culture which was significantly high (*P* < 0.01).

The parameters for obtaining plaque that most likely contain *H. pylori* are unknown. Studies showed that *H. pylori* are not uniformly distributed in the mouth. Prospective studies are needed to identify the best methods to ensure that *H. pylorus* is not missed because of improper sampling. The subgingival microbiota in patients with gingival problems provides a significant and persistent Gram-negative bacterial challenge to the host. These organisms and their products have direct access to the circulation via the ulcerated epithelium. Proper maintenance of oral hygiene will reduce this subgingival pathogenic microbiota and it in turn might control the systemic disease. Whether dental plaque represents a common ecological niche for this organism has not been established, but these findings should encourage the systematic investigation of this site and other possible sources, to gain further insight into the epidemiology of *H. pylori*.

## CONCLUSION

*H. pylori,* a Gram-negative, microaerophilic motile organism which may be present in the oral cavity as a consequence of gastric reflex. Further studies are needed to confirm whether removal of plaque can cause any change in the recurrence rate. Demonstration of oral carriage of *H. pylori*, transient or permanent, may have immediate applications with recommendations to prevent person-to-person transmission via the oral-oral route.

Eradication of oral *H. pylori* by local medication or oral hygiene procedures would rely on the precise identifications of its oral ecological niche. Future directions into usage of polymerase chain reaction and serology test in detection of *H. pylori* will gain further insight into oral *H. pylori*, and it would offer the potential for the noninvasive test for the infection.
